# The Protective Effect of Baicalin against UVB Irradiation Induced Photoaging: An In Vitro and In Vivo Study

**DOI:** 10.1371/journal.pone.0099703

**Published:** 2014-06-20

**Authors:** Jia-an Zhang, Zhi Yin, Li-wen Ma, Zhi-qiang Yin, Yan-yan Hu, Yang Xu, Di Wu, Felicia Permatasari, Dan Luo, Bing-rong Zhou

**Affiliations:** Department of Dermatology, the First Affiliated Hospital of Nanjing Medical University, Nanjing, Jiangsu, P. R. China; Roswell Park Cancer Institute, United States of America

## Abstract

**Objective:**

This study was aimed to evaluate the anti-photoaging effects of baicalin on Ultraviolet B (UVB)-induced photoaging in the dorsal skin of hairless mice and premature senescence in human dermal fibroblasts.

**Methods:**

We established *in vivo* and *in vitro* photoaging models by repeated exposures to UVB irradiation. By HE staining, masson staining, immunohistostaing and real-time RT-PCR, we analyzed epidermal thickness, collagen expression and the mRNA and protein levels of type I collagen, type III collagen, interstitial collagenase (MMP-1 and MMP-3) in UVB exposed dorsal mice skin. The aging condition in human dermal fibroblasts was determined by senescence-associated β-galactosidase (SA-β-gal) staining. Cell viability was determined using the Cell Counting Kit-8 (CCK-8). The G1 phase cell growth arrest was analyzed by flow cytometry. The senescence-related protein levels of p16^INK-4a^, p21^WAF-1^, and p53 and protein levels of phosphorylated histone H2AX were estimated by Western blotting.

**Results:**

Topically application of baicalin treatment reduced UVB-induced epidermal thickening of mouse skin and also result in an increase in the production of collagen I and III, and a decrease in the expression of MMP-1 and MMP-3. Compared with the UVB-irradiated group, we found that the irradiated fibroblasts additionally treated with baicalin demonstrated a decrease in the expression of SA-β-gal, a increase in the cell viability, a decrease in the G1 phase cell proportion, a downregulation in the level of senescence-associated and γ-H2AX proteins. However, Baicalin had no difference in the normal fibroblasts without UVB irradiation and long-term Baicalin incubation of UVB-SIPS fibroblasts gave no effects on the cell proliferation.

**Conclusions:**

Taken together, these results suggest that baicalin significantly antagonizes photoaging induced by UVB *in vivo* and *in vitro*, indicating the potential of baicalin application for anti-photoaging treatment.

## Introduction

Skin aging involves intrinsic and extrinsic processes. Environmental factors, primarily ultraviolet (UV) light, cause extrinsic skin aging. Although there are various etiologies of skin photoaging, common points include less dermal type I and III collagen expression [1]. The predominant form of collagen in dermis is type I, followed by small amounts of type III [2]. Type I collagen is characterized by thick fiber that confer stiffness and resistance to perform a crucial function in maintaining the structure of dermis. Whereas collagen type III is characterized by thin fiber that present the resiliency of skin. Collagen fibers arrange parallel to skin surface and are responsible for the high tensile strength and resiliency of skin. The degradation of type I and III fibrillar collagens is initiated by matrix metalloproteinases-1 (MMP-1) and MMP-3 respectively, which belongs to the matrix metalloproteinases (MMPs), a large family of zinc-dependent endo-proteases with a broad range of substrate specificities and the capacity of degrading all extracellular matrix proteins. Fibroblasts regulate production and degradation of the extracellular matrix and make multiple cytokines and glycoproteins. Fibroblasts exposed to UV reduce collagen by both promoting its degradation and interfering with its production [3]. Other studies have reported early senescent changes that were confirmed by measuring β-galactosidase activity, p53, p21 and p16 expressions were detected when human fibroblasts were exposed properly to UVB [4]. In previous reports, fibroblasts could be photoaged by UVB *in vitro* and *in vivo* and these experimental models were proven to be applicable in various skin aging studies [5], [6], [7], [8].

Baicalin is the predominant flavonoid isolated from the roots of *Scutellaria lateriflora Georgi* (Huang Qin). It has been reported that this compound exhibits many different pharmacological activities. Baicalin has displayed beneficial effects on several diseases model such as hypoxia/reoxygenation caused cardiomyocytes injury [9], hepatic cytotoxicity [10], iron-overloaded mouse [11], rheumatoid arthritis [12] and so on. Furthermore, recent studies have confirmed the photoprotective effect of baicalin against acute and multiple UVB-induced photodamage [13], [14], [15], [16], and this effect is thought to be associated with reduction of oxidative stress [17]. However, whether baicalin can protect human dermal fibroblasts form UVB irradiation induced premature senescence remains to be clarified.

In this study, the anti-photoaging effects of baicalin were evaluated in isolated dermal skin fibroblasts and the skin of hairless mice. For in vitro studies, the effects of baicalin on expressions of β-galactosidase activity, protein levels of p53, p21, p16 and γ-H2AX, production in skin fibroblasts after UVB irradiation were investigated. For in vivo studies, young mice were pretreated with UVB irradiation to induce photoaging. The expression of for MMP-1, MMP-3, collagen-1 and collagen-3 were quantitatively measured from the nontreated and baicalin-treated mouse skin using immpunostaining and real-time RT-PCR methods.

## Materials and Methods

### In vivo study

#### Animals and UV light source

Female C57BL/6 mice (aged 6–8 weeks, weighing 20–25 g) were obtained from Chinese Academy of Science, Shanghai SLAC Laboratory Animal CO. LTD and maintained in a pathogen-free barrier facility at Nanjing Medical University, The study complied with standards for the Care and Use of Laboratory Animals (Laboratory Animal Center of Nanjing Medical University). Experimental Animal Center and all experiment protocols were approved with respect to animal experimentation and care of animals under study, All animal protocols were approved by the Institutional Animal Care and Use Committee (IACUC) of Nanjing Medical University (China) (Permit Number: 20110521). The source of UVB was BLE-1T158 (Spectronics Corp., Westbury, NY, USA). A Kodacel filter (TA401/407, Kodak, Rochester, USA) was used to block wavelengths of less than 290 nm (ultraviolet C). The UVB dosage was quantified using a Waldmann UV meter (model no. 585100: Waldmann Co., VS-Schwenningen, Germany).

#### Treatments of animals

Purified Baicalin was purchased from the National Institute for the Control of Pharmaceutical and Biological Products (Beijing, China). Female C57BL/6 mice were divided into following six groups. Five mice in the first group did not receive any treatment and served as a control. Five mice in the second group received a topical treatment of baicalin (1 mg/cm^2^ skin area/mouse/100 µL acetone) on their dorsal skin. Five mice in the third group received UVB treatment. The mice in the fourth group received a topical application of 100 µL acetone followed by UVB irradiation (2 hours following acetone treatment). The mice in the fifth group received a topical application of baicalin (0.5 mg/cm^2^ skin area/mouse/100 µL acetone) followed by UVB irradiation (2 hours following baicalin treatment). The mice in the sixth group received a topical application of baicalin (1 mg/cm^2^ skin area/mouse/100 µL acetone) followed by UVB (2 hours following baicalin treatment). The irradiation intensity represented as the minimal erythemal dose (MED) was set at 1 MED during the first 2 weeks (60 mJ/cm^2^), and was elevated to 2 MED (120 mJ/cm^2^) in the 3^rd^ week, to 3 MED (180 mJ/cm^2^) in the 4^th^ week and to 4 MED (240 mJ/cm^2^) during the 5^th^–8^th^ weeks of the experiment. The total irradiated UVB volume was approximately 115 MED (6.9 J/cm^2^). Studies were performed at 24 h after the last UVB exposure. For histopathology examination and immunohistochemical analysis, the back skin biopsies were placed in 10% phosphate-buffered formalin. For real-time RT-PCR detection, the fat was removed and the back skin biopsies were stored in liquid nitrogen.

#### Histology

After treatment with polyester wax, the skin samples were sliced into 6-mm thicknesses. The sliced sections were treated with haematoxylin and eosin (H&E) and Masson Trichrome staining solutions. Throught issue evaluations, the thickness of the epidermal layer and presence of collagen fibres were observed. The thickness of the epidermal layer was calculated by measuring at 40 different sites from each section, and the mean value of the thickness of the epidermal layer for each group was used for the comparison. The amounts of collagen fibers in Masson Trichrome stained sections were measured using an image analysis computer program (BMI plus software, BumMi Universe Co.), and expressed as the percentage area occupied by each fiber in the upper dermis.

#### Immunohistochemical analysis

Antigen retrieval was performed by immersing in 10 mM citrate buffer and microwave oven for 20 min. The slides were incubated overnight at 4°C either with rabbit mAb against collagen I (Abcam, MA, USA) in a 1 ∶ 100 dilution or rabbit mAb against collagen III (Abcam, MA, USA) in a 1 ∶ 100 dilution or rabbit mAb against MMP-1 (Abcam, MA, USA) in a 1 ∶ 100 dilution or rabbit mAb against MMP-3 (Abnova, Taiwan) in a 1 ∶ 75 dilution. Negative controls were incubated in the same dilution of matched normal immunoglobulin G. After extensive washing with PBS for 20 min twice, the slides were incubated for 25 min with biotinylated anti-mouse secondary antibody (Dako, Ely, UK) at room temperature. After washing in PBS, a LSAB2 kit based on the streptavidin–biotin–peroxidase reaction was used. Peroxidase activity was then measured using a 3,3-diaminobenzidine (DAB) substrate. All slides were counterstained with Harris haematoxylin and dehydrated in a graded series of alcohols and xylene, and coverslips were applied with permount mounting media under standard conditions.

#### Real-time RT-PCR detection

The expressions of procollagen types I and III, MMP-1, MMP-3 genes were determined according to the protocol of KeyGen Biotech Co., Ltd., Nanjing, Jiangsu, China. Total RNA was extracted from skin samples by using TRIzol (Invitrogen, USA). cDNA was synthesized from the isolated RNA using SuperScript III Reverse Transcriptase (Keygen, China). PCR was performed on ABI Prism 7700 Sequence Detector (Applied Biosystems). Specific primers were listed in table 1. For data analysis, the ΔΔCt method was used. For each gene, fold-change was calculated as the difference in gene expression between two groups. A positive value indicated gene up-regulation and a negative value indicated gene down-regulation. The results were expressed as mean ±SD of 3 independent experiments.

**Table 1:** Primers used in the real-time RT-PCR amplification of the mouse procollagen types I and III, MMP-1, MMP-3 genes and GAPDH mRNAs.

Gene	Forward Primer (5′-3′)	Reverse Primer (5′-3′)	Amplicon Size (Bp)
Procollagen I	5′- CAGGCAAACCTGGTGAACA -3′	5′- CTCGCCAGGGAAACCTCT -3′	89
Procollagen III	5′- CTGGACCCCAGGGTCTTC -3′	5′- GACCATCTGATCCAGGGTTTC -3′	78
MMP-1	5′- GCTAACCTTTGATGCTATAACTACGA -3′	5′- TTTGTGCGCATGTAGAATCTG -3′	75
MMP-3	5′- CTCCAACCGTGAGGAAAATC -3′	5′- CATGGAATTTCTCTTCTCATCAAA -3′	110
GAPDH	5′- TGTTGCCATCAATGACCCCTT -3′	5′- CTCCACGACGTACTCAGCG -3′	202

### In vitro study

#### Cell Culture

Normal human skin samples were obtained from circumcisions in accordance with the ethical committee approval process of Jiangsu Provincial People's Hospital, Nanjing, Jiangsu, China. The study was approved by the Local Ethics Committees of the First Affiliated Hospital with Nanjing Medical University, Nanjing, Jiangsu, China. Written informed consent was obtained from all participants in this study. Specimens were sterilized in 70% ethanol, minced, and incubated in Dulbecco's modified Eagle medium (DMEM) supplemented with 10% fetal bovine serum and 1% penicillin–streptomycin in an atmosphere of 5% CO_2_ at 37°C. Dermal HDFs normally grew from the explants after 5–7 days. The cells from passages 8 to 11 were used in this study.

#### Ultraviolet B (UVB) Irradiation

UVB-stressed cells were irradiated at a subcytotoxic dose of 10 mJ/cm^2^ twice a day for 5 days [6]. Before UVB irradiation, the medium was removed and covered with phosphate buffered saline (PBS). UVB irradiation was delivered by using a Philips TL 20W/12 (Eindhoven, The Netherlands), a fluorescent bulb emitting 280–320 nm wavelength with a peak at 313 nm. Irradiation output was monitored by using a Waldmann UV-meter (Waldmann, Villigen-Schwenningen, Germany).

#### Group Divisions and Treatments

The cells were divided into six groups: 1: control group: no treatments; 2: UVB-SIPS group: the cells received UVB irradiation; 3: baicalin group: the cells were incubated with 25 µg/ml of baicalin without receiving UVB irradiation; 4, 5, 6: UVB-SIPS+baicalin groups: the cells received UVB irradiation, serum-starved for 2 days, and then were incubated with baicalin at three different concentrations (6.25, 12.5, and 25 µg/ml, respectively) for 2 days.

#### Cell proliferation assay

Cell proliferation was assayed using a CCK-8 Kit (Beyotime Institute of Biotechnology, Nantong, Jiangsu, China). In brief, 100 µl of cells (2×10^3^ cells/well) were transferred into 96-well plates after digestion with trypsin, and five parallel wells were used for each treatment. After attachment, the cells were subjected to the different treatments, and then cultured for 24 h in a 5% CO_2_ incubator at 37°C. Subsequently, 10 µl of CCK-8 was added to each well, and the cells were cultured for another 3 h. Cell density was determined by measuring the absorbance at 450 nm using a Varioskan Flash (Thermo Scientific, USA).

#### β-galactosidase staining for detection of senescent cells

To measure one of the biomarkers of senescence, senescence-associated β-galactosidase (SA-β-gal) staining was performed. The cells were fixed in 2% formaldehyde/0.2% glutaraldehyde, rinsed with PBS and incubated at 37°C with fresh SA-β-gal stain solution, which is composed of 1 mg of 5-bromo-4-chloro-3-indolyl β-D galactoside (X-Gal) per mL (stock = 20 mg of dimethylformamide per mL), 40 mM citric acid, sodium phosphate(pH 6.0),5 mM potassium ferrocyanide, 150 mM NaCl and 2 mM MgCl_2_.

#### Flow cytometry for detection of G1 phase-cell percentage

To determine whether UVB-stressed HDFs exhibit cell growth arrest, cell-cycle analysis with flow cytometry was performed. HDFs were fixed with 70% alcohol, washed twice with PBS, digested with RNase and stained with propidium iodide (PI). A flow cytometer (FAC-Scan, BD, NJ, USA) was used to gather data and images, to analyze the cell cycle, and to calculate the percentage of cells in the G1 phase.

#### Western Blotting for detection of p16^INK–4a^, p21^WAF–1^, p53 and γ-H2AX

To investigate the level of senescence-related proteins (p16^INK–4a^, p21^WAF–1^, p53 and γ-H2AX proteins), Western Blotting analysis was performed. The cells were lyzed in 62.5 mM Tris–HCl (pH 6.8) containing 2% w/v SDS, and the protein concentration was determined by the Pierce BCA assay (Thermo Fisher Scientific, Rockford, IL, USA). Mercaptoethanol and bromophenol blue were added to make the final composition equivalent to the LaemmLi sample buffer. Samples were fractionated using SDS-polyacrylamide gel electrophoresis (SDS-PAGE) and blotted onto Immobilon-P membrane (Millipore, Billerica, MA, USA). Rabbit anti-mouse HRP (1∶1,000 dilution) and goat anti-rabbit HRP (1∶1,000 dilution) were used as secondary antibodies (Biotime, Haimen, China). Antibody binding was visualized via Pierce ECL reagents (Thermo Fisher Scientific). We used mouse monoclonal antibodies against p53 and p21^WAF–1^ (Cell Signaling Technology, California, USA), rabbit monoclonal antibody against p16^INK–4a^ (sc-601) (Bethyl Laboratories, USA) and phospho-H2AX (Millipore, MA, USA), and monoclonal anti-actin antibody as a control. Quantification of protein bands was established by Band-Scan software (PROZYME, San Leandro, California).

#### Statistical analysis

Statistical analysis was performed by using SPSS for Windows version 16.0 (SPSS, Chicago, IL, USA). Data are expressed as the mean ± SD for each group. The statistical analysis for the statistical in different groups was performed using a non-paired t-test. A p-value of less than 0.05 was considered statistically significant.

## Results

### Baicalin suppresses UVB-induced epidermal thickening in mouse skin

**Figure 1:**
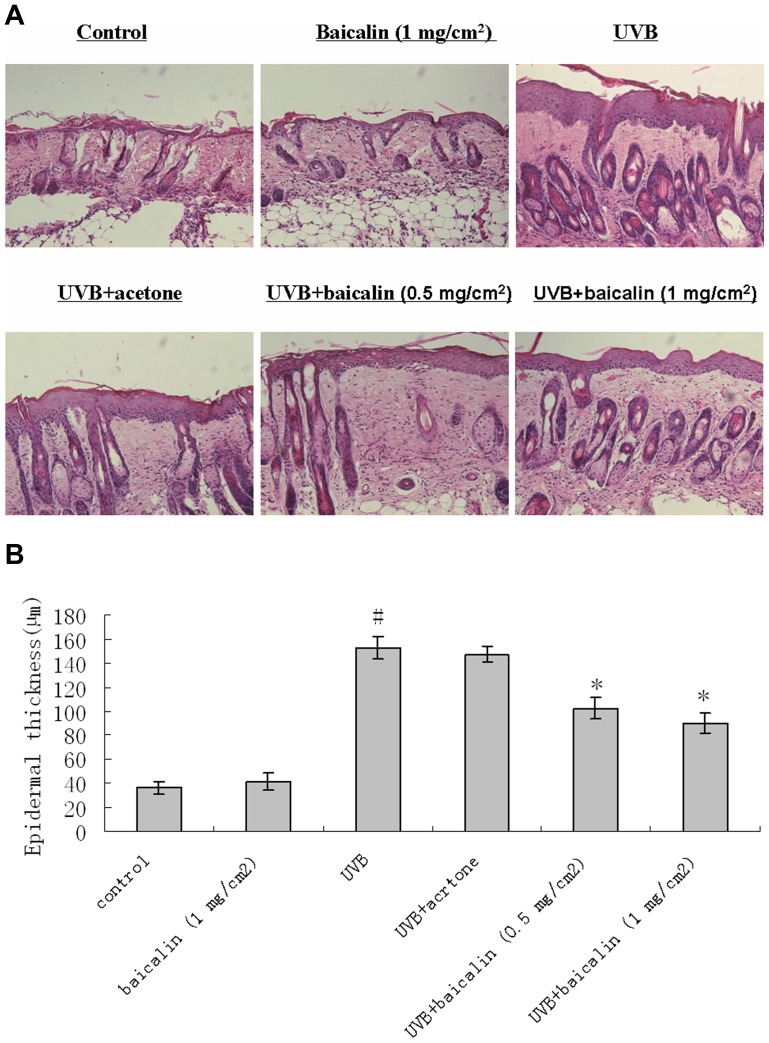
Baicalin prevents UVB induction of increased mouse epidermal thickness. (A) After mice were treated as described for methods, the dorsal skin was excised, sectioned, mounted onto slides, and stained with hematoxylin and eosin for measurement of epidermal thickness. Images are representative of results from 5 tissue samples. Original magnification ×40. (B) Bars represent the mean thickness (µm) of epidermis from 5 animals (40 measurements/section). Results are shown as means±SD (n = 5). The symbol (#) indicates a significant difference (p<0.05) between the control group and the UVB-irradiated group. Asterisks (*) indicate significant differences of p<0.05, respectively, between the baicalin-treated and non-treated groups of irradiated mice.

Because epidermal thickening is a major biomarker of photoaging [18], we evaluated the effect of baicalin on UVB-induced epidermal thickening. In a quantitative analysis, hematoxylin and eosin staining demonstrated that UVB irradiation induced 4.23 folds increase in epidermal thickness (p<0.05 vs. the non-irradiated control group; n = 5). Topically applied baicalin (0.5 or 1 mg/cm^2^ skin area/mouse/100 µL acetone) decreased the amount of UVB-induced epidermal thickening respectively (p<0.05 vs. the irradiated group; n = 5) (Fig. 1)

### Baicalin protects against UVB -induced dermal collagen fiber loss

**Figure 2:**
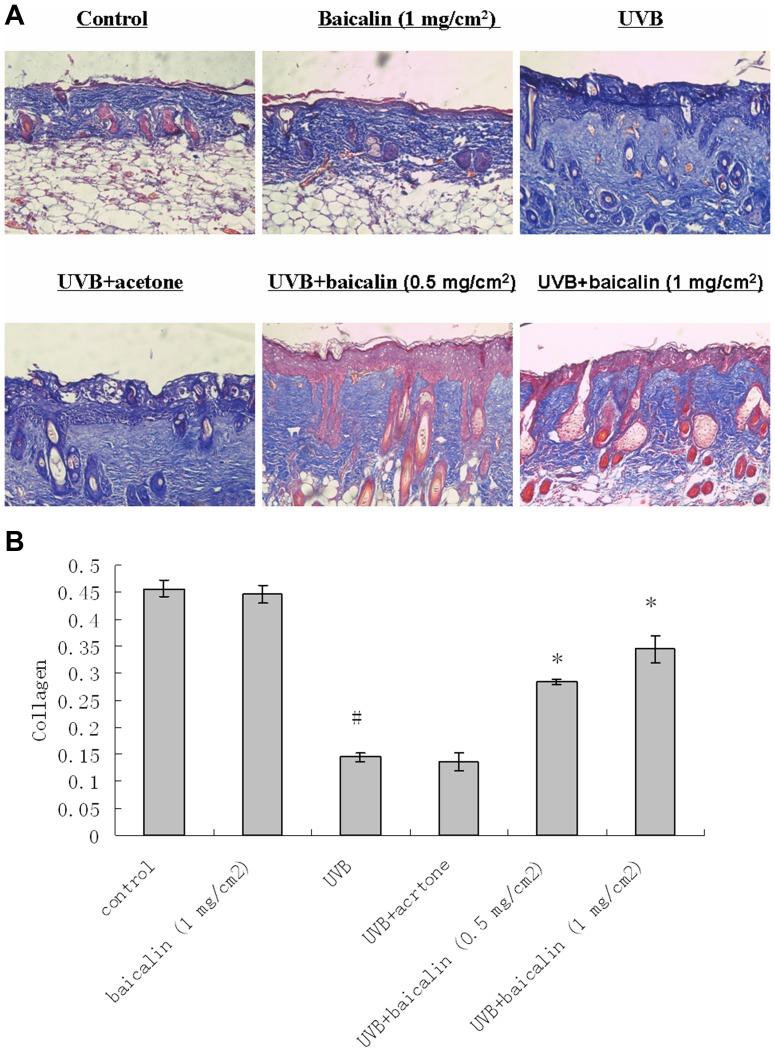
Baicalin protects against UVB irradiation-induced dermal collagen fiber loss. (A) Mouse skin samples were fixed in 10% formalin and embedded in paraffin. Each section (6 mm) was stained with masson-trichrome for collagen fibers. Images are representative of results from 5 tissue samples. Original magnification ×40. (B) Bars represent the mean density value of the collagen fiber, which was stained in blue color, from 5 animals (10 measurements/section). Results are shown as means±SD (n = 5). The symbol (#) indicates a significant difference (p<0.05) between the control group and the UVB-irradiated group. Asterisks (*) indicate significant differences of p<0.05, respectively, between the baicalin-treated and non-treated groups of irradiated mice.

We found that collagen levels were decreased significantly in UVB-radiated mice by about 68.17% (p<0.05, N = 5, Fig. 2), compared with control mice, but baicalin alone had no effect. However, in baicalin-radiated mice skin, baicalin at two different dosages augmented the UVB-induced collagen compared with UVB-radiated mice.

### Baicalin prevented UVB-induced decrease in type I and III collagen in dorsal mice skin

**Figure 3:**
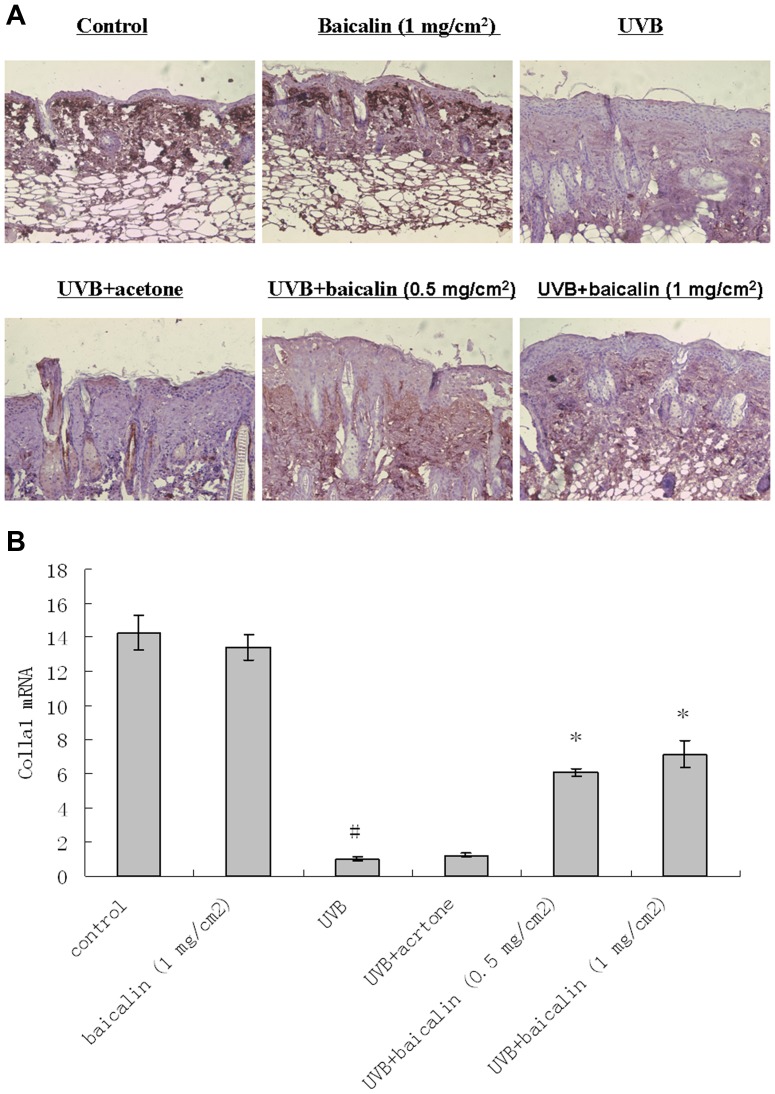
Baicalin protects against UVB irradiation-induced type I collagen fiber loss. (A) Skin specimens of dorsal trunk were harvested for immuno-staining using primary antibodies of procollagen type I collagen. Images are representative of results from 5 tissue samples. Original magnification ×40. (B) Total RNA was extracted from biopsied skin samples of different groups. Type I procollagen mRNA was determined by real-time RT-PCR analysis. Results are shown as means±SD (n = 5). The symbol (#) indicates a significant difference (p<0.05) between the control group and the UVB-irradiated group. Asterisks (*) indicate significant differences of p<0.05, respectively, between the baicalin-treated and non-treated groups of irradiated mice.

**Figure 4:**
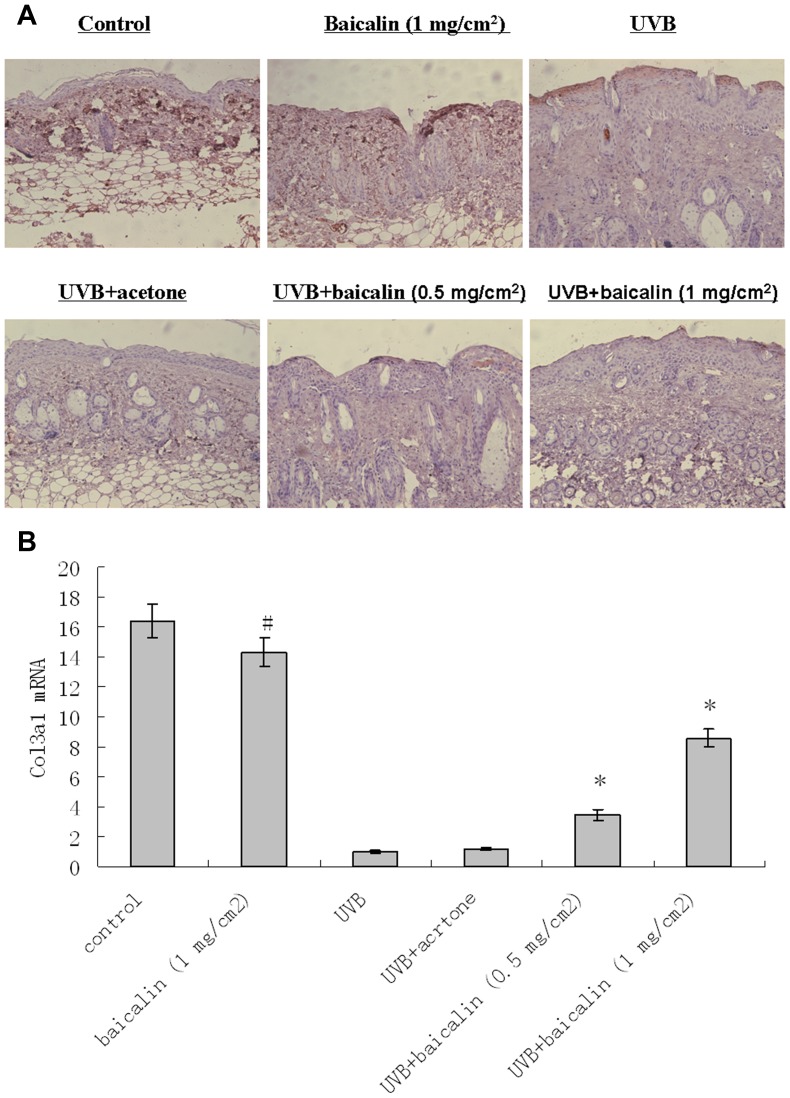
Baicalin protects against UVB irradiation-induced type III collagen fiber loss. (A) Skin specimens of dorsal trunk were harvested for immuno-staining using primary antibodies of procollagen type III collagen. Images are representative of results from 5 tissue samples. Original magnification ×40. (B) Total RNA was extracted from biopsied skin samples of different groups. Type III procollagen mRNA was determined by real-time RT-PCR analysis. Results are shown as means±SD (n = 5). The symbol (#) indicates a significant difference (p<0.05) between the control group and the UVB-irradiated group. Asterisks (*) indicate significant differences of p<0.05, respectively, between the baicalin-treated and non-treated groups of irradiated mice.

Immunohistochemistry revealed that UVB decreased type I and III collagen expression throughout the dermis (Fig. 3a and 4a). Baicalin-pretreated skin demonstrated greater intracellular procollagen staining in dermal fibroblasts, compared with vehicle-pretreated skin, after UV irradiation, indicating that the Baicalin pretreatment counteracted the downregulating effects of UV on type I and III collagen (Fig. 3a and 4a). However, in unirradiated skin, baicalin-treated skin demonstrated no obviously impaction on type I and III collagen expression compared with the vehicle-treated control. For real-time RT-PCR results of type I and type III procollagens, the similar modulation tendency to those in the histological and immunochemistry pictures was observed i.e. there was a significant statistical difference of mRNA expressions between UVB treated and UVB+baicalin treated groups (p<0.05, Fig. 3b and 4b), which implied Baicalin could prevented UVB-induced decrease mRNA expression of type I and III procollagen.

### Baicalin prevented UVB-induced induction of MMP-1 and 3 in dorsal mice skin

**Figure 5:**
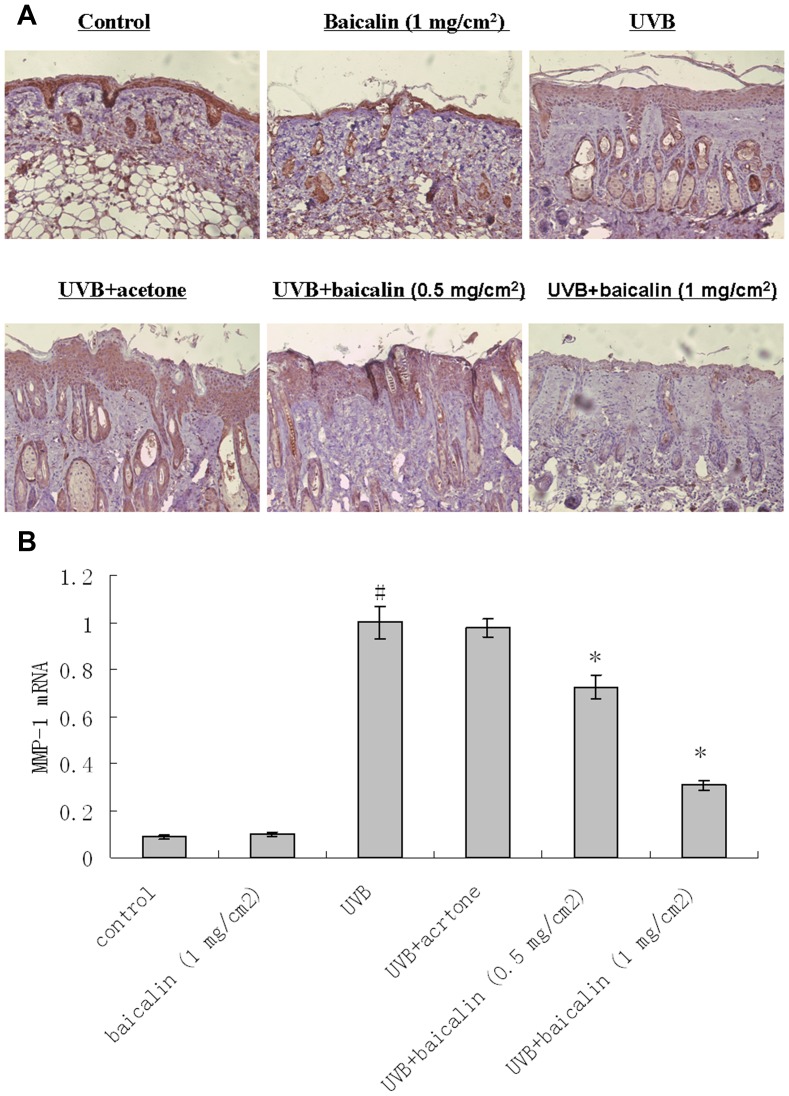
Baicalin protects against UVB irradiation-induced amplification of MMP-1 expression. (A) Skin specimens of dorsal trunk were harvested for immuno-staining using primary antibodies of MMP-1. Images are representative of results from 5 tissue samples. Original magnification ×40. (B) Total RNA was extracted from biopsied skin samples of different groups. MMP-1 mRNA was determined by real-time RT-PCR analysis. Results are shown as means±SD (n = 5). The symbol (#) indicates a significant difference (p<0.05) between the control group and the UVB-irradiated group. Asterisks (*) indicate significant differences of p<0.05, respectively, between the baicalin-treated and non-treated groups of irradiated mice.

**Figure 6:**
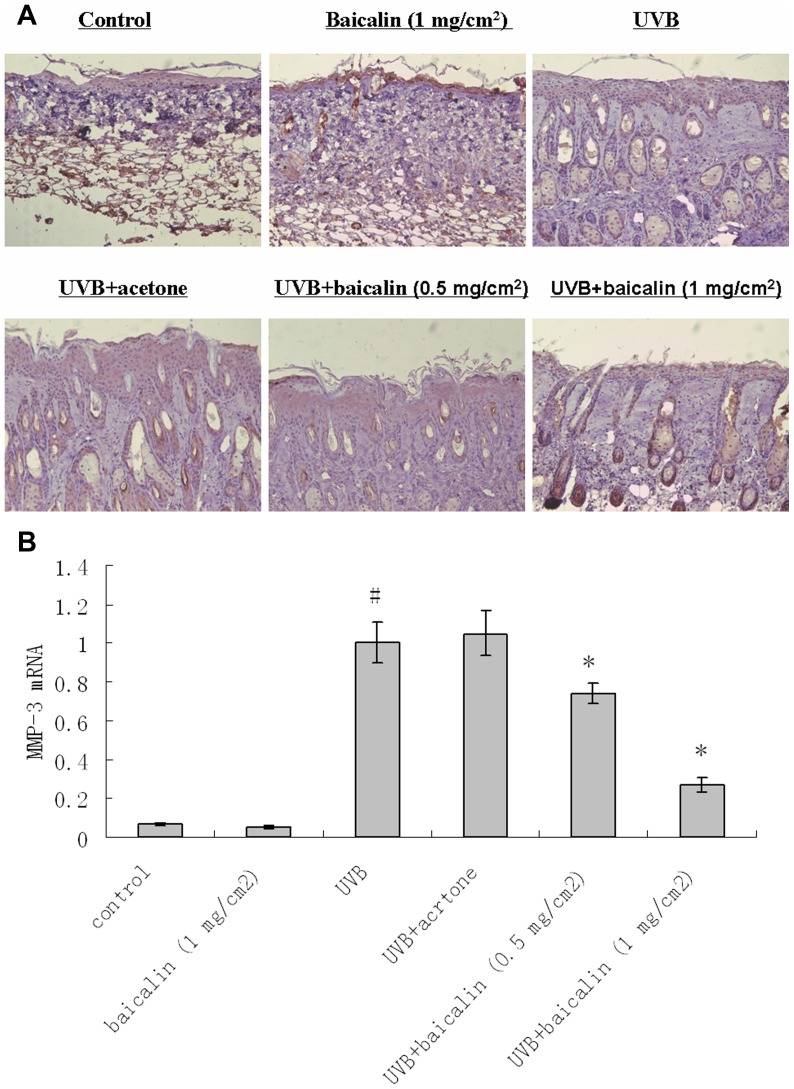
Baicalin protects against UVB irradiation-induced amplification of MMP-3 expression. (A) Skin specimens of dorsal trunk were harvested for immuno-staining using primary antibodies of MMP-3. Images are representative of results from 5 tissue samples. Original magnification ×40. (B) Total RNA was extracted from biopsied skin samples of different groups. MMP-3 mRNA was determined by real-time RT-PCR analysis. Results are shown as means±SD (n = 5). The symbol (#) indicates a significant difference (p<0.05) between the control group and the UVB-irradiated group. Asterisks (*) indicate significant differences of p<0.05, respectively, between the baicalin-treated and non-treated groups of irradiated mice.

Immunohistochemistry revealed that UVB induced amplification of MMP-1 and 3 expression throughout the skin, especially in epidermis (Fig. 5a and 6a). Baicalin-pretreated skin demonstrated decreased MMP-1 and 3 staining in skin, compared with vehicle-pretreated skin, after UV irradiation, indicating that the Baicalin pretreatment counteracted the upregulating effects of UVB on MMP-1 and 3 (Fig. 5a and 6a). For real-time RT-PCR results of MMP-1 and 3, the similar modulation tendency to those in the histological and immunochemistry pictures was observed i.e. there was a significant statistical difference of mRNA expressions between UVB treated and UVB+baicalin treated groups (p<0.05, Fig. 5b and 6b), which implied baicalin could prevented UVB-induced mRNA expression of MMP-1 and 3.

### Baicalin protects HDFs against UVB-SIPS induced impaired cell viability

**Figure 7:**
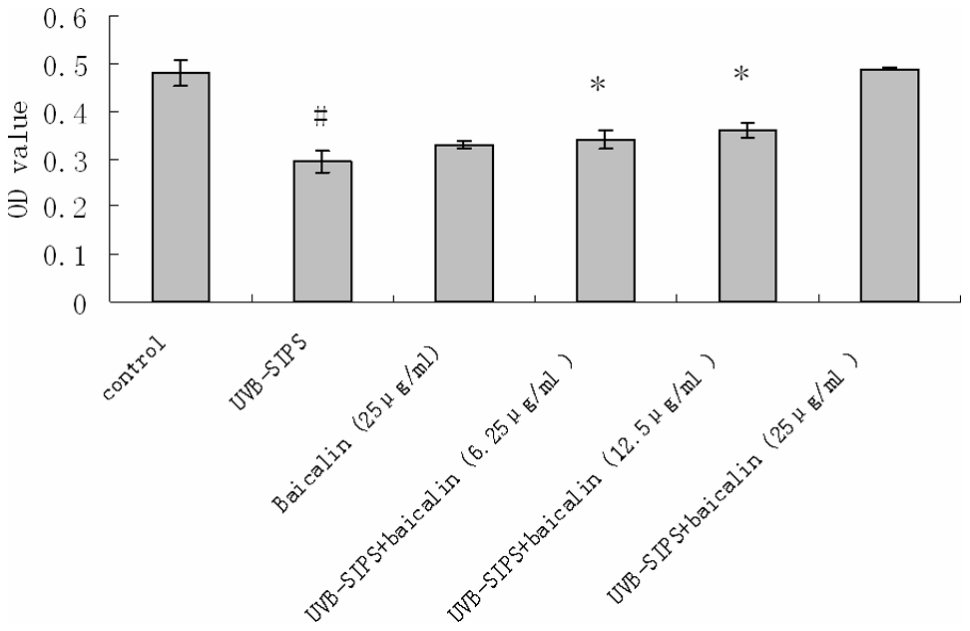
Baicalin protects cultured HDFs against UVB-SIPS induced impaired cell viability. Baicalin exerted a protective effect in a concentration- dependent manner. HDFs were irradiated with UVB at a subcytotoxic dose of 10 mJ/cm^2^ twice a day for 5 days, and then cultured with 6.25, 12.5 and 25 µg/ml baicalin. 24 hours after last treatment, the cell viability was assayed by using a CCK-8 assay kit. Values are given as mean ± SD (n = 5). The symbol (#) indicates a significant difference (p<0.05) between the control group and the UVB-SIPS group. Asterisks (*) indicate significant differences of p<0.05, respectively, between the baicalin-treated and UVB-SIPS cells.

Compared with the control group, the cell viability of the UVB-SIPS group was significantly decreased (P<0.05) (Fig. 7). The cell viability in the UVB-irradiated groups treated with 6.25 µg/ml, 12.5 µg/ml, and 25 µg/ml of baicalin compared to that of the UVB-SIPS group showed significant increases in a dose-dependent manner, whilst there seemed to no obvious reduction in the cell viabilit of the baicalin group compared to the control group (Fig. 7).

### Baicalin decreased the percentage of SA-β-gal positive cells in UVB-SIPS fibroblasts

**Figure 8:**
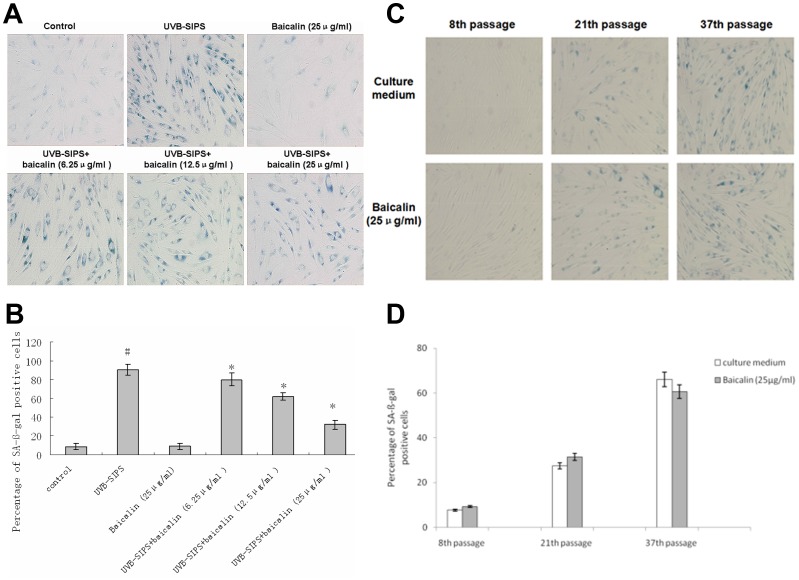
Baicalin protects cultured HDFs against UVB induced premature senescence, while has no protection for the replicative senescence. Fibroblasts were stained for SA-β-gal and photographed under 100× magnifications (A) (C) The percentage of SA-β-gal positive cells is shown in Figure 8. (B) (D) Statistical analysis was carried out with the Student's t-test. The symbol (#) indicates a significant difference (p<0.05) between the control group and the UVB-SIPS group. Asterisks (*) indicate significant differences of p<0.05, respectively, between the baicalin-treated and UVB-SIPS cells.

The cells in the UVB-SIPS group became obviously enlarged, flattened, and irregular compared to the control group (Fig. 8a). There was a 10.2-fold increase of positive cells in the UVB-SIPS group compared to the control group. The percentage of positive cells in UVB-SIPS group was 90.52%, while it was 8.91% in the control group. The percentage of *SA-β-gal* positive cells in the UVB-irradiated groups treated with 6.25, 12.5 and 25 µg/ml baicalin was significantly reduced compared to that in the UVB-SIPS group in a dose-dependent manner. The percentage of positive cells in the UVB+6.25 µg/ml, 12.5 µg/ml, and 25 µg/ml of baicalin were 80.13%, 62.44%, and 32.88%, respectively. There was no significant difference in the percentage of positive cells between control group and baicalin group (Fig. 8b). However, the positive expression of SA-β-gal showed no difference in normal fibroblasts treated with or without baicalin, even for a long term (Fig. 8c and 8d).

### Baicalin decreased G1 phase cell proportion in UVB-SIPS fibroblasts

**Figure 9:**
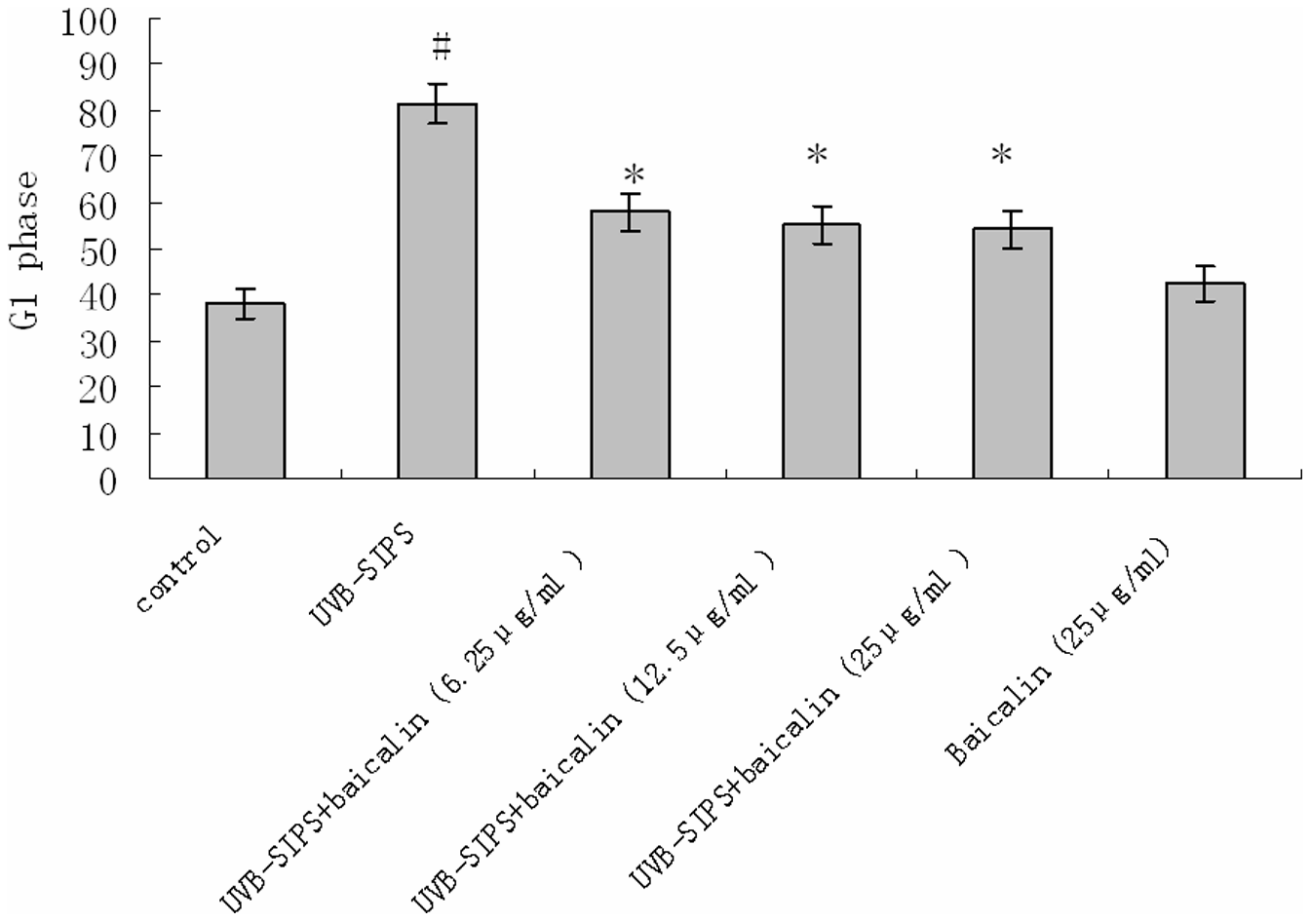
Baicalin protects cultured HDFs against UVB-SIPS induced G1 arrest. The percentage of cells in G1 blockage after treatment in each group. Data are expressed as the mean of at least three determinations. Statistical analysis was carried out with Student's t-test. The symbol (#) indicates a significant difference (p<0.05) between the control group and the UVB-SIPS group. Asterisks (*) indicate significant differences of p<0.05, respectively, between the baicalin-treated and UVB-SIPS cells.

The results showed that UVB-stressed HDFs were blocked mostly in the G1 phase of the cell cycle. Compared with the control group (from 38.1% to 81.5%), the G1 phase cell proportion of the UVB-SIPS group was significantly higher (P<0.05) (Fig. 9). The G1 phase cell proportions in the UVB-irradiated groups treated with 6.25, 12.5 and 25 µg/ml baicalin compared to that of the UVB-SIPS group showed significant decreases in a dose-dependent manner (from 81.5% to 57.97%, 55.2% and 54.12%, respectively). There seemed to be no obvious reduction in the G1 phase cell proportion of the baicalin group compared to the control group (from 42.64% to 38.1%) (Fig. 9).

### Baicalin decreased the level of p16^INK-4a^, p21^WAF-1^, and p53 proteins in the UVB-SIPS fibroblasts

**Figure 10:**
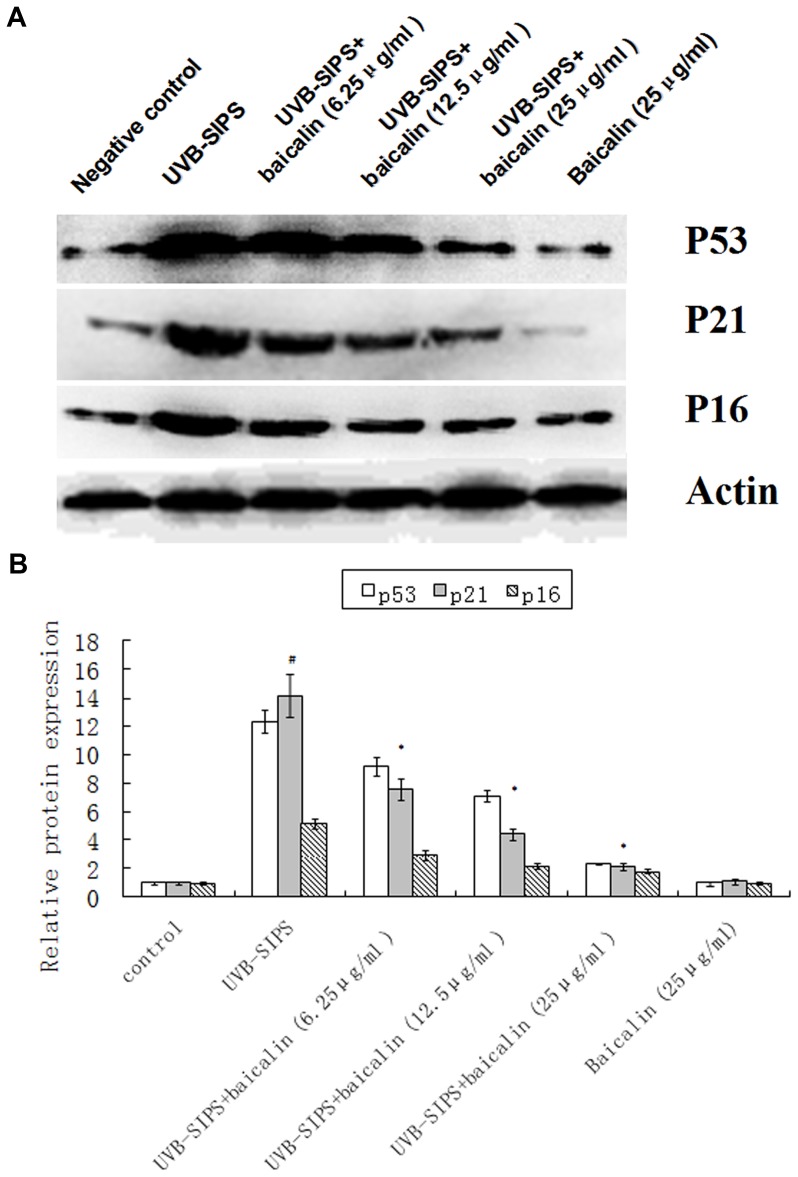
Baicalin protects cultured HDFs against UVB-SIPS induced expression of senescence-related proteins. (A) Expression of p-P53, p21^WAF-1^, and p16^INK-4a^ after UVB stress treated with or no with baicalin were detected by Western blotting. (B) Band-Scan software was used to analyze each band in the gray scale. The ratio of gray scale values represent the ratio of amount of protein of interest/actin, and we calculated the relative ratio of every treatment group/control group. The results represented the mean relative ratio of triplicates. These results were corresponding to the features of senescence, as described above. Statistical analysis was carried out with Student's t-test. The symbol (#) indicates a significant difference (p<0.05) between the control group and the UVB-SIPS group. Asterisks (*) indicate significant differences of p<0.05, respectively, between the baicalin-treated and UVB-SIPS cells.

The levels of p16^INK-4a^, p21^WAF-1^, and p53 proteins in the UVB-SIPS group showed 5.2-, 14.2-, and 12.3-fold increase, respectively, compared to the control group. The levels of p16^INK-4a^, p21^WAF-1^, and p53 proteins in the UVB-irradiated groups treated with 6.25, 12.5 and 25 µg/ml baicalin decreased significantly, respectively, compared to those of the UVB-SIPS group. There were no significant changes in the level of p16^INK-4a^, p21^WAF-1^, and p53 protein in the baicalin group compared to that in the control group (Fig. 10a and 10b).

### Baicalin caused significant a dose-dependent decrease in the level of γ-H2AX proteins in the UVB-SIPS fibroblasts

**Figure 11:**
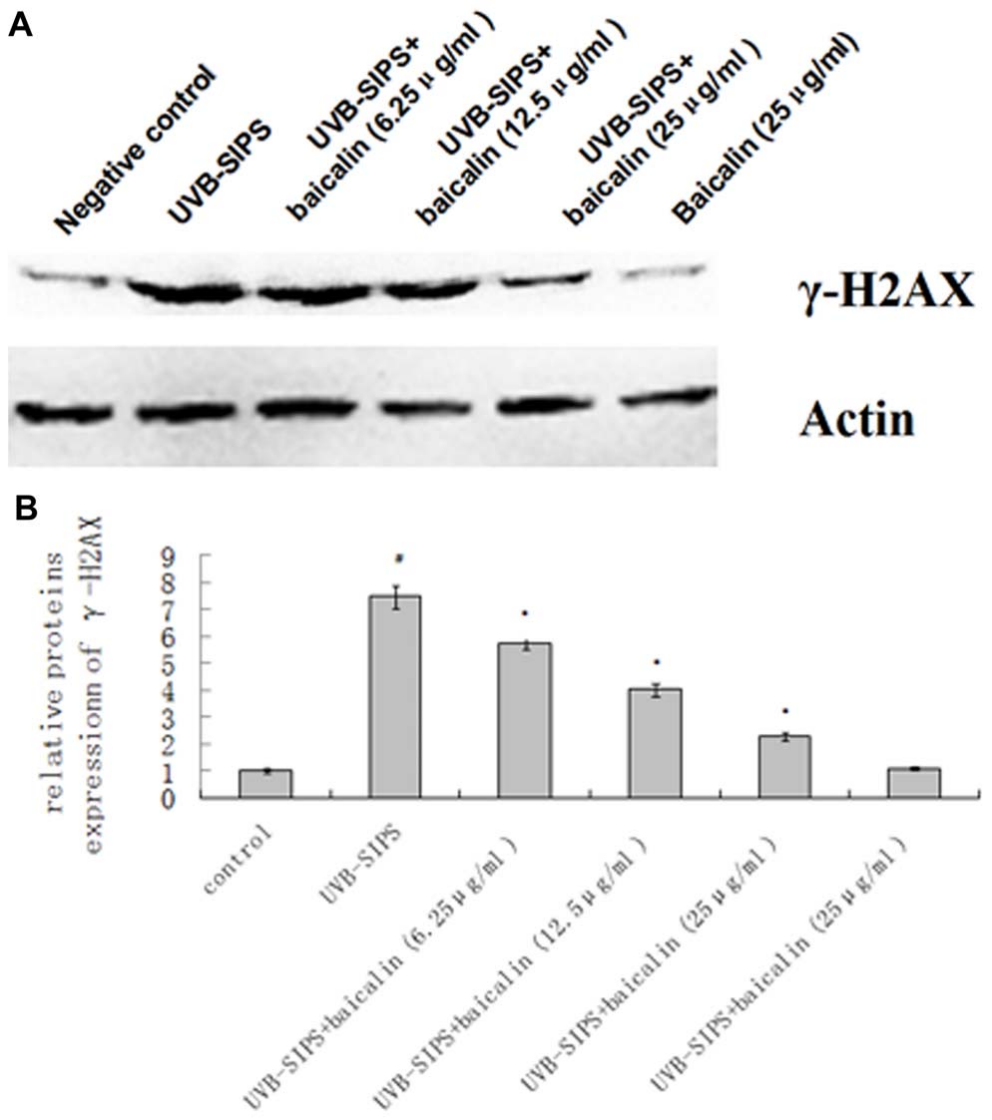
Baicalin protects cultured HDFs against UVB-SIPS induced expression of γ-H2AX proteins. (A) Expression of γ-H2AX after UVB stress treated or untreated with baicalin were detected by Western blotting. (B) Band-Scan software was used to analyze each band in the gray scale. The ratio of gray scale values represents the ratio of amount of protein of interest/actin, and we calculated the relative ratio of every treatment group/control group. The results represented the mean relative ratio of triplicates. Statistical analysis was carried out with Student's t-test. The symbol (#) indicates a significant difference (p<0.05) between the control group and the UVB-SIPS group. Asterisks (*) indicate significant differences of p<0.05, respectively, between the baicalin-treated and UVB-SIPS cells.

There was a 7.5-fold increase in the levels of γ-H2AX proteins in the UVB-SIPS group compared to the control group. The levels of γ-H2AX in the UVB-SIPS groups treated with 6.25, 12.5 and 25 µg/ml baicalin was significantly decreased compared to that of the UVB-SIPS group. There was no significant difference in the levels of γ-H2AX in the baicalin group compared to that in the control group (Fig. 11).

### Long-term Baicalin incubation of UVB-SIPS fibroblasts gave no effects on the cell proliferation

**Figure 12:**
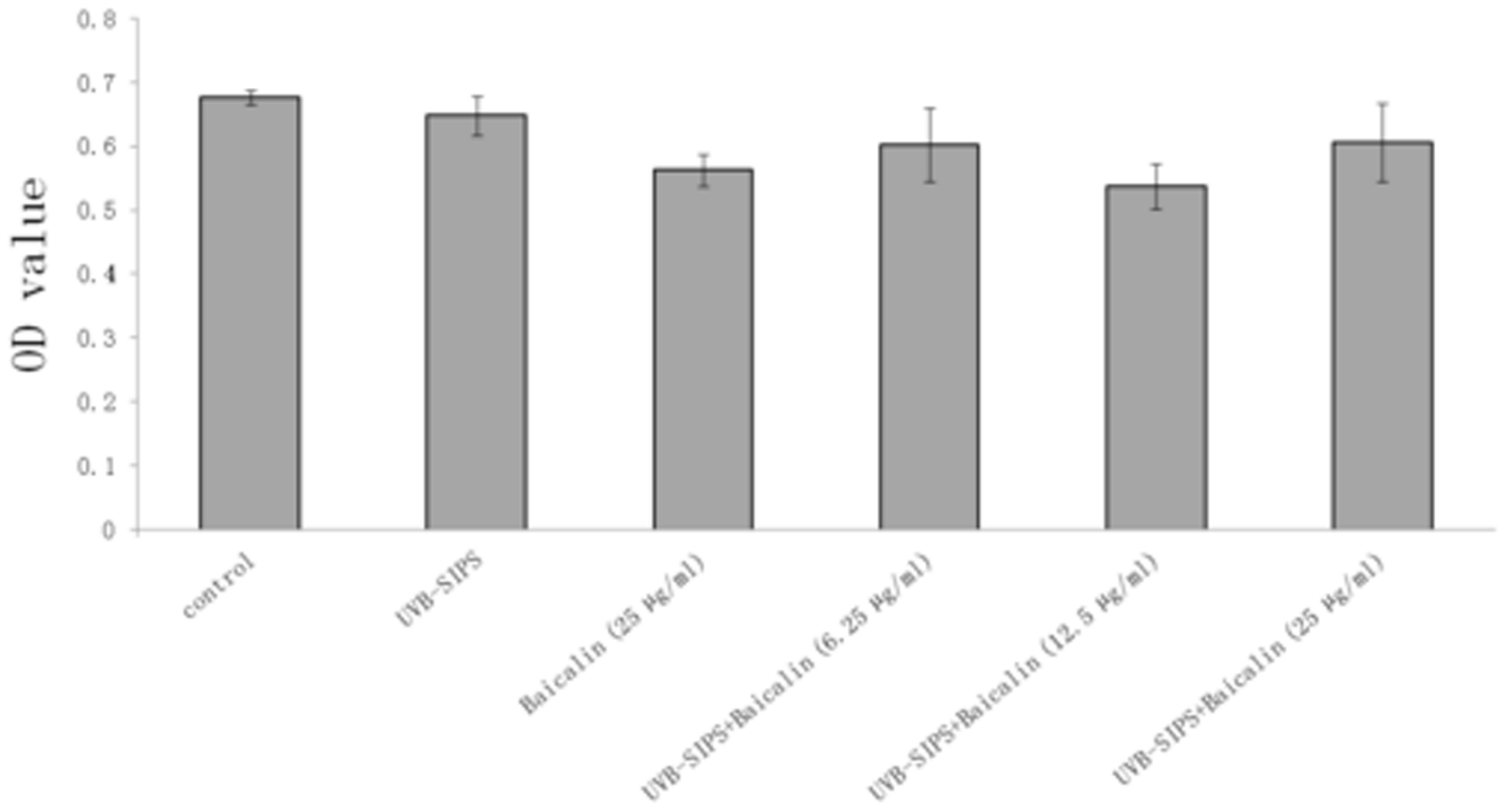
Baicalin does not display any promotion on the long term proliferation of fibroblasts after UVB irradiation. HDFs were irradiated with UVB at a subcytotoxic dose of 10/cm2 twice a day for 5 days, and then cultured with 6.25, 12.5 and 25 µg/ml baicalin. With the culture over 8 weeks, the cell viability was assayed by using a CCK-8 assay kit. Values are given as mean ± SD (n = 5).

With a long term culture of fibroblasts over 8 weeks, the cell proliferation in the UVB-SIPS group treated with 6.25 µg/ml, 12.5 µg/ml, and 25 µg/ml of baicalin showed no difference with the UVB-SIPS group, whilst there seemed to no obvious reduction in the cell proliferation of the baicalin group compared to the control group (Fig. 12).

## Discussion

One promising strategy for the prevention of photoaging is the targeting and suppression of collagen degradation using natural phytochemicals. As natural products, these phytochemicals most likely are relatively harmless and possess a variety of beneficial properties. The antioxidant and anti-photodamage properties of baicalin, a naturally occurring flavonoid, have been the subject of much study [14], [15], [16], [17]. However, the specific therapeutic properties and actions of baicalin in the prevention of photoaging are unknown. In the present study, we investigated the potential anti-photoaging effects of baicalin on UVB-exposed mouse dorsal skin and human dermal fibroblasts.

Histological studies have shown that photoaging of skin is associated with increased epidermal thickness and alterations in connective tissue organization [19], [20]. Similarly, we found that chronic UVB irradiation of mouse skin induced epidermal thickening and topically applied baicalin inhibited these effects of UVB. Another key condition of skin photoaging associated with atrophy of the dermal connective tissue is the destruction of extracellular matrix (ECM) components, in particular collagen fibers. Consist with previous reports, in present results, we found Collagen levels also decreased significantly after 10 weeks of UVB irradiation. In Baicalin+UVB-radiated mice, baicalin treatment attenated the UV-induced decrease in collagen. These effects of baicalin may resulting in relief of photoaging by UV radiation. Several MMPs have been implicated in photoaging and aging process [21]. In particular, UV light is known to induce the expression of MMP-1 and MMP-3 in normal mice epidermis in vivo [22]. Also, UV irradiation impairs ongoing collagen synthesis, primarily through the down-regulation of types I and III procollagen expression [23]. Our results demonstrate that the chronic exposure of mouse skin to UVB light induces the expression of MMP-1, which is involved in the degradation of type I collagen fragments generated by collagenase. According to our results, topically application with baicalin (+UV group) had strong anti-photoaging effects. Our results suggest that the mechanism underlying the anti-photoaging effects of baicalin in UVB-exposed hairless mice involves the inhibition of collagen degradation and increased collagen synthesis. As the generation of reactive oxygen species (ROS) by UV irradiation [24] is considered to be a major reason to activate AP-1, which is the transcription factor stimulates the transcription of matrix metalloproteinase (MMP) genes [25]. The anti-oxidative properties of baicalin has been proved by our previous investigation. In this consideration, we hypothesized that the documented anti-oxidative effects of baicalin may be one reasonable explanation for the its anti-photoaging capacity.

Once cells have entered senescence, they undergo a dramatic change in morphology: their volume increases and they lose their original shape with a flattened cytoplasm. It is confirmed that the positive expression of SA-β-gal increases in aging individuals, so SA-**β**-gal can serve as one kind of aging-related biological markers [26]. In accordance to this, we found that there was a large increase in the percentage of SA-**β**-gal positive cells in the UVB-SIPS group, suggesting that UVB irradiation specifically induces premature senescence in HDFs. Meanwhile, baicalin at a dose of 25 µg/ml did not increase the percentage of SA-**β**-gal positive cells in the non-UVB treatment group, indicating that baicalin did not enforce the cells into senescence and give no cytotoxicity on HDFs. Compared with the UVB-SIPS group, we found that baicalin decreased the percentage of SA-**β**-gal positive cells with increasing dosage, suggesting that baicalin possesses a dose-dependent anti-senescence capacity.

Excessive generation of ROS by UV irradiation causes a range of DNA damages, including DNA strand breaks (DSBs), DNA-protein cross-links, deletion mutations [27], [28]. γ-H2AX, which has been identified as an early event after DSBs formation, is considered the most sensitive assay for DSBs detection [29]. In our study, we found that irradiation of UVB resulted in an increased expression ofγH2AX. Treatment with baicalin reduced the UVB-induced γH2AX expression.Senescence makes the cells cease to proliferate, and this is due to growth arrest [26]. A drastically decreased proliferative potential of HDFs in UVB-SIPS group was observed after 5 exposures to UVB [6]. HDFs in UVB-SIPS group were blocked mainly in the G1 phase of the cell cycle as were senescent ones, significantly higher than that in control group [6]. Compared with the UVB-SIPS group, UVB-SIPS+baicalin groups displayed a remarkable reduction in the percentage of cells in G1 arrest with increasing dose of baicalin. This result suggests that baicalin can stimulate the proliferation of photoaged fibroblasts. It is well established that the p53-p21-pRb and p16-pRb signaling pathways are involved in aging and photoaging. Researches have elucidated the initial molecular events responsible for most of the typical manifestations of aging and photoaging, revealing an intellectual framework that links the two processes [30]. Active p53 is known to trigger overexpression of p21^WAF-1^. Both p21^WAF-1^ and p16^INK-4a^ are cyclin dependent kinase inhibitors that block the cell cycle in G1 phase [31]. In accordance with the cell cycle analysis results, we found that baicalin decreased protein levels of p16^INK-4a^, p21^WAF-1^, and p53, in a dose-dependent manner. UVB irradiation increases the percentage of cells in G1 phase, while baicalin can decrease the percentage of cells in G1 phase by inhibiting the expression of p16, p21, and p53. This indicates one mechanism by which baicalin rescued the UVB-SIPS fibroblasts from growth arrest.

Senescence has commonly been considered a tumor-suppressive mechanism [32]. Usually anti-senescence mechanism has the potential to cause malignancies. Our results showed that baicalin has anti-photoaging capacity. However, baicalin had no effection on the fibroblasts without UVB irradiation, even for a long term. Meanwhile, long-term Baicalin incubation of UVB-SIPS fibroblasts gave no effects on the cell proliferation. Based on the results, baicalin seems not to show potential malignant transformation under UV in the models tested.

In conclusion, baicalin has positive effects on UVB-SIPS human dermal fibroblasts by inducing cell proliferation via decreasing senescence-related proteins, increasing collagen production, and decreasing collagen degradation. The results obtained in the present study indicate that baicalin can play a role in anti-UVB-induced premature senescence, which can be used for future clinical usage.
